# Altered temporal connectivity and reduced meta-state dynamism in adolescents born very preterm

**DOI:** 10.1093/braincomms/fcad009

**Published:** 2023-02-07

**Authors:** Katri Lahti, Sirkku Setänen, Victor Vorobyev, Anna Nyman, Leena Haataja, Riitta Parkkola

**Affiliations:** Department of Pediatric Neurology, University of Turku and Turku University Hospital, PO Box 52, FI-20521, Turku, Finland; Department of Adolescent Psychiatry, University of Turku and Turku University Hospital, Kunnallissairaalantie 20, rak4, 3.krs PL52, 20520 Turku, Finland; Department of Pediatric Neurology, University of Turku and Turku University Hospital, PO Box 52, FI-20521, Turku, Finland; Department of Diagnostic Radiology, University of Turku, Kiinamyllynkatu 4-8, FI- 20520 Turku, Finland; Department of Social Research, 20014 University of Turku, Turku, Finland; Children’s Hospital, University of Helsinki, PO Box 22 (Stenbäckinkatu 11), 00014 Helsinki, Finland; Department of Diagnostic Radiology, University of Turku, Kiinamyllynkatu 4-8, FI- 20520 Turku, Finland

**Keywords:** preterm behavioural phenotype, resting-state magnetic resonance imaging, functional network connectivity, meta-state dynamism, adolescent neuropsychiatry

## Abstract

Adolescents born very preterm have an increased risk for anxiety, social difficulties and inattentiveness, i.e. the ‘preterm behavioural phenotype’. The extreme end of these traits comprises the core diagnostic features of attention and hyperactivity disorders and autism spectrum disorder, which have been reported to show aberrant dynamic resting-state functional network connectivity. This study aimed to compare this dynamism between adolescents born very preterm and controls. A resting-state functional magnetic resonance imaging was performed on 24 adolescents born very preterm (gestational age <32 weeks and/or birth weight ≤1500 g) and 32 controls born full term (≥37 weeks of gestation) at 13 years of age. Group-wise comparisons of dynamic connectivity between the resting-state networks were performed using both hard clustering and meta-state analysis of functional network connectivity. The very preterm group yielded a higher fraction of time spent in the least active connectivity state in hard clustering state functional network connectivity, even though no group differences in pairwise connectivity patterns were discovered. The meta-state analysis showed a decreased fluidity and dynamic range in the very preterm group compared with controls. Our results suggest that the 13-year-old adolescents born very preterm differ from controls in the temporal characteristics of functional connectivity. The findings may reflect the long-lasting effects of prematurity and the clinically acknowledged ‘preterm behavioural phenotype’.

## Teaser

Lahti *et al*. report diminished dynamics in brain functional connectivity studied with the functional MRI in resting state as a possible sign of long-lasting consequences of very premature birth on brain connectivity in 13-year-old adolescents compared to the term-born peers.

## Graphical Abstract

**Graphical abstract:**
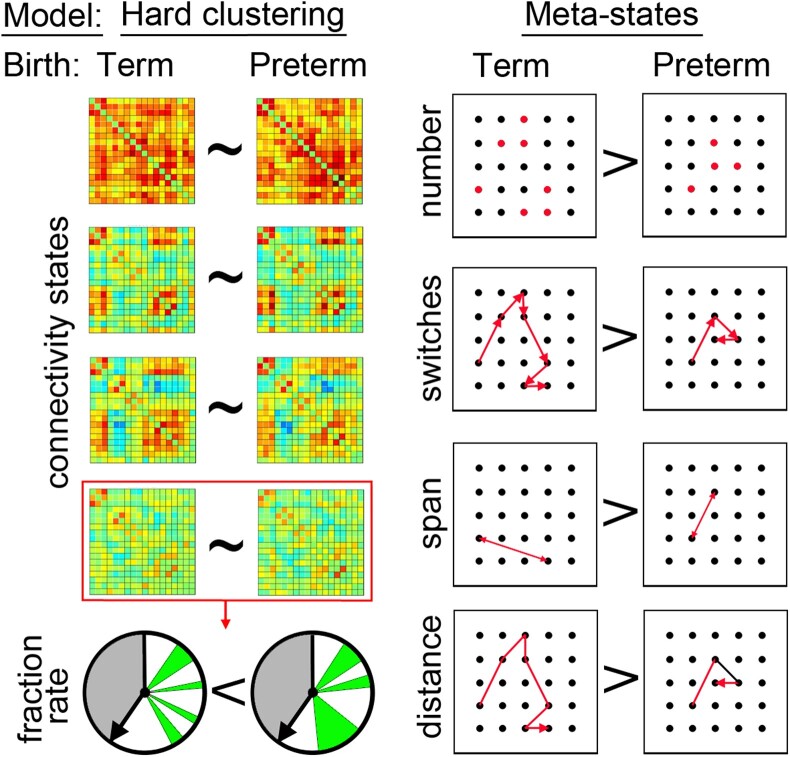

## Introduction

Very preterm (VPT) birth (gestational age <32 weeks and/or birth weight ≤1500 g) increases the risks for developmental difficulties in areas such as learning, language and motor skills.^1^ Besides these, children born VPT characteristically shows a typical ‘preterm behavioural phenotype’ characterized by inattention, anxiety and social difficulties.^2^ The spectrum of these symptoms varies from mild to extreme, which includes the diagnoses of attention deficit hyperactivity disorder, especially the attention deficit subtype, and autism spectrum disorder. These traits have wide-ranging effects on wellbeing, social relations and quality of life and they persist in adulthood.^1-4^

Resting-state functional MRI (rsfMRI) is an imaging technique that measures fluctuations in the blood oxygen level dependent (BOLD) signal across the brain in the absence of tasks. Independent component analysis (ICA) is capable of extracting distinct networks (independent components) represented by spatial maps and related time courses of functional activity from the functional MRI (fMRI) data. The spatial maps of the independent components may represent various resting-state networks (RSNs), while the activity time courses can be used to explore correlative structure between the RSNs termed as functional network connectivity (FNC). The FNC can be analysed both in static and dynamic ways.^5^ The static FNC method assumes that the connectivity is constant over the scanning time, while the dynamic approaches take into account fluctuations of the brain connectivity, which might be more informative.^6^

Therefore, in this study, we applied ICA to extract RSNs and analysed dynamic FNC between different RSNs using two different approaches. The first one interprets connectivity for each short period (time window) as the representation of one out of a few stable connectivity states thus reducing the data to a sequence of these reoccurring states over scan time. This approach is termed here as the hard clustering dynamic FNC method; it has been detailed in Allen *et al.*^6^ The second approach avoids such data reduction. Instead, it expresses each unique windowed connectivity situation as a weighted sum of the states where the weight vector constitutes a meta-state from a vast variety of other possible meta-states, thus allowing a high-dimensional analysis of dynamic FNC.^7^ While the hard clustering method can reveal a group difference in connectivity strength between each pair of RSNs and state changes in time, the meta-state method provides no data about pair correlations, but produces some metrics about fluidity and range of the meta-states ‘visited’ by subjects.^6,8,9^

The previous rsfMRI studies utilizing the static FNC method have shown controversial results when comparing VPT-born adolescents and controls.^10-15^ When compared to controls, preterm-born children did not differ in terms of static FNC at the age of eight years. However, the same participants underwent a new scanning at the age of 16 and at that age, it was found that preterm-born teenagers displayed greater relative connectivity compared to controls.^10,14^ The patterns between network and within-network connectivity have been shown to be altered differently. For example, in adolescents born VPT, the connectivity has been shown to be increased between the central executive network, salience network and sensorimotor network and decreased within the central executive network. Especially the central executive network and salience network are of high importance regarding keeping and shifting attention.^15^ In addition, altered connectivity of the amygdala has been shown to be associated with altered behavioural profiles in parent reports in VPT-born young adults.^16^

In a previous study, it has been shown, that preterm birth might alter the FNC between RSNs and that these alterations could be associated with the executive function deficits seen in attention deficit hyperactivity disorder or autism spectrum disorder.^17^ We aimed to compare the temporal FNC measures between 13-year-old adolescents born VPT and controls. We hypothesized that the adolescents born VPT would show an altered brain functionality due to prematurity and that the alterations might resemble those seen in adolescents with attention deficit hyperactivity disorder or autism spectrum disorder.^18,19^ To our knowledge, there are no dynamic FNC studies in adolescents born VPT.

## Materials and methods

### Subjects

**Figure 1:**
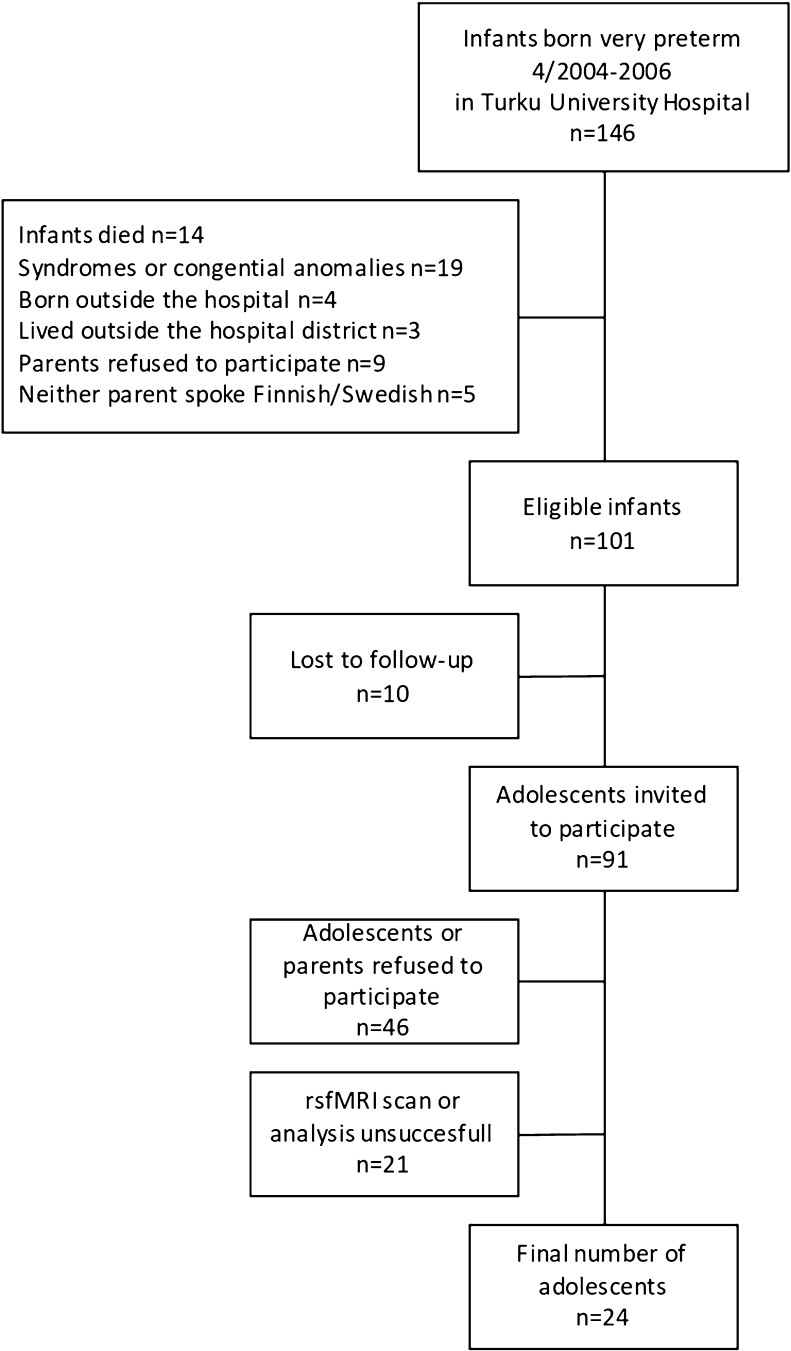
A flow chart of the adolescents born VPT is included in this study.

This study is part of the multidisciplinary PIPARI study (The Development and Functioning of Very Low Birth Weight Infants from Infancy to School Age) of VPT infants (gestational age <32 weeks/birth weight ≤1500 g) born in Turku University Hospital in 2001–2006 and controls born full term (gestational age ≥37 weeks) in the same hospital 2003–2004.^20,21^ For this study, we invited the adolescents born VPT in 4/2004–2006. The flow chart is shown in Fig. 1. The full-scale intelligence quotient (FSIQ) of the adolescents born VPT was measured at the age of 11 with the Wechsler Intelligence Scale for Children-IV^22,23^ as a part of the longitudinal follow-up study.

The recruitment protocol, including the inclusion and exclusion criteria of the healthy controls, has previously been described in detail.^24^ A total of 97 controls were invited, 38 of whom agreed to attend. The reasons for dropping out were either refusal of the adolescent to participate in the imaging study or the parents withdrawing from the study in an earlier phase of this longitudinal follow-up. The parental education level did not differ between adolescents born VPT and controls.

The Ethics Review Committee of the Hospital District of Southwest Finland approved the study protocol in 2012. The adolescents and their parents provided separate informed consents. Together with the informed consent, the families willing to participate were asked to report any diagnoses given to and medications affecting the central nervous system used by the adolescent.

### Data collection and analysis

#### MRI data collection

MRI data were acquired with a 3.0 T Philips Ingenuity scanner (Philips Medical Imaging, Best, the Netherlands) equipped with a 32-channel head coil. The BOLD series (single-shot echo-planar imaging, repetition time 2000 ms, echo time 20 ms, flip angle 75, 35 axial slices, matrix 80 × 80, slice thickness 4 mm, gap 0 mm and in-plane resolution 3 × 3 mm) were acquired in ascending order in a single session. For each participant, 234 volumes were collected during a 7.8 min fMRI sequence. In addition, a structural high-resolution (1 mm^3^ voxel) T1-weighted image (set of sagittal slices with a repetition time of 8.1 ms and an echo time of 3.7 ms) was acquired.

The comprehensive MR images were clinically assessed by an experienced neuroradiologist (R.P.) and the classification of the possible brain pathology is described in detail in Lind *et al*.^25^ Head movement was minimized by stabilizing the head with a pillow and providing hearing protection in form of ear plugs and headphones. Before the resting-state series, the subjects were instructed to relax with their eyes closed without thinking about anything special.

#### Preprocessing of structural and functional MRI data

The preprocessing was performed with free software packages implemented in MATLAB (Mathworks Inc., Sherbom, MA, USA). In order to account for potential systematic structural differences between adolescent and adult brains,^26^ a sample-specific brain template was created using the Computational Anatomy Toolbox, CAT12 (http://www.neuro.uni-jena.de/cat12-html). First, each structural volume was segmented and normalized to Montreal Neurological Institute brain space using affine transformation and age-specific tissue probability maps obtained with the Template-O-Matic TOM8 software.^27^ The affinely normalized images were checked for sample homogeneity, resulting in the selection of 30 subjects per group. The selected data were used to create a customized sample-specific template using a diffeomorphic algorithm, DARTEL.^28^ Then, the original individual structural images were registered again to the space of the sample-specific template with DARTEL to record individual deformation fields.

The fMRI data preprocessing was performed with Statistical Parametric Mapping (SPM12, http://www.fil.ion.ucl.ac.uk/spm). Six starting volumes were discarded to allow T1 equilibration and subject state stabilization, resulting in 228 volumes for data processing. The fMRI data of each subject were corrected for slice-timing difference using the middle slice as a reference and corrected for head motion by co-registering scans to the realigned mean. Subjects with mean between-scan relative motion exceeding 0.2 mm in translation or 0.2° in rotation were rejected, after which 24 adolescents were born VPT and 32 controls remained. The resulting groups differed neither in translation (*P* = 0.95) nor rotation (*P* = 0.35) according to the Mann–Whitney U-test. In addition, the framewise displacement was calculated to be used later as a confound in the connectivity analyses. Its median values and interquartile intervals were 0.184 and 0.11 for the controls and 0.217 and 0.13 for the preterm-born group with no group difference (*P* > 0.05) according to the Mann–Whitney U-test. Functional images were co-registered to a scalp-stripped structural image for each subject using rigid-body transformation and then spatially transformed to the space of the sample-specific structural template using corresponding deformation fields, resliced to a 3 mm cubic voxel and smoothed with a 6 mm isotropic Gaussian kernel.

#### Denoising of functional MRI data

For additional cleaning of artefacts, the voxel time series were despiked^29^ and further cleaned using the Conn toolbox^30^ by regressing out linear trend, motion-related signal and first-order derivatives (12 parameters); regressor for outlier scans (exceeding 4 SD in global signal), mean white matter and cerebrospinal fluid signals and their 16 principal components.^31^ Finally, the time series were band-pass (0.01–0.1 Hz) filtered.

#### ICA

Spatial ICA was performed on the preprocessed and denoised fMRI data with the group ICA for the fMRI toolbox (GIFT v3.0b; http://trendscenter.org/software)^8^ after removing mean intensity over time for each voxel within the brain mask. We opted to use a low-order model to obtain easily recognizable networks and to avoid excessive splitting of the networks into smaller subnetworks. Given that 20–30 components appear to provide a representative characterization of large-scale networks, 25 independent components were extracted.^32^ Data dimensionality was reduced to 35 principal components, followed by the decomposition of the group-reduced matrix into 25 spatially independent components by the Infomax ICA algorithm, repeated 20 times in Icasso to ensure stability.^33^ Individual independent components (spatial maps and time courses) were back-reconstructed using the group ICA. The main maxima for each spatial map were defined using the results of one sample *t*-tests performed with SPM12. Components with maxima in the grey matter, frequency maxima within the 0.01–0.1 Hz range and spatial maps having only negligible overlap with non-grey matter areas were considered as RSNs.^34^ The remaining components were rejected from further analysis as related to noise factors.

Among the 25 independent components, 18 were identified as RSNs, belonging to the following RSN domains: default mode network, salience network, posterior memory network, fronto-parietal network, ventral attention network, dorsal attention network, network with maxima in superior precuneus, angular and frontal cortices, visual network, sensory-motor network, auditory network, subcortical network, cerebellar network and limbic network (Supplementary Fig. 1). The locations of the main maxima for each RSN are listed in the Supplementary Table 1.

#### Extraction of windowed FNC correlation matrices

**Figure 2:**
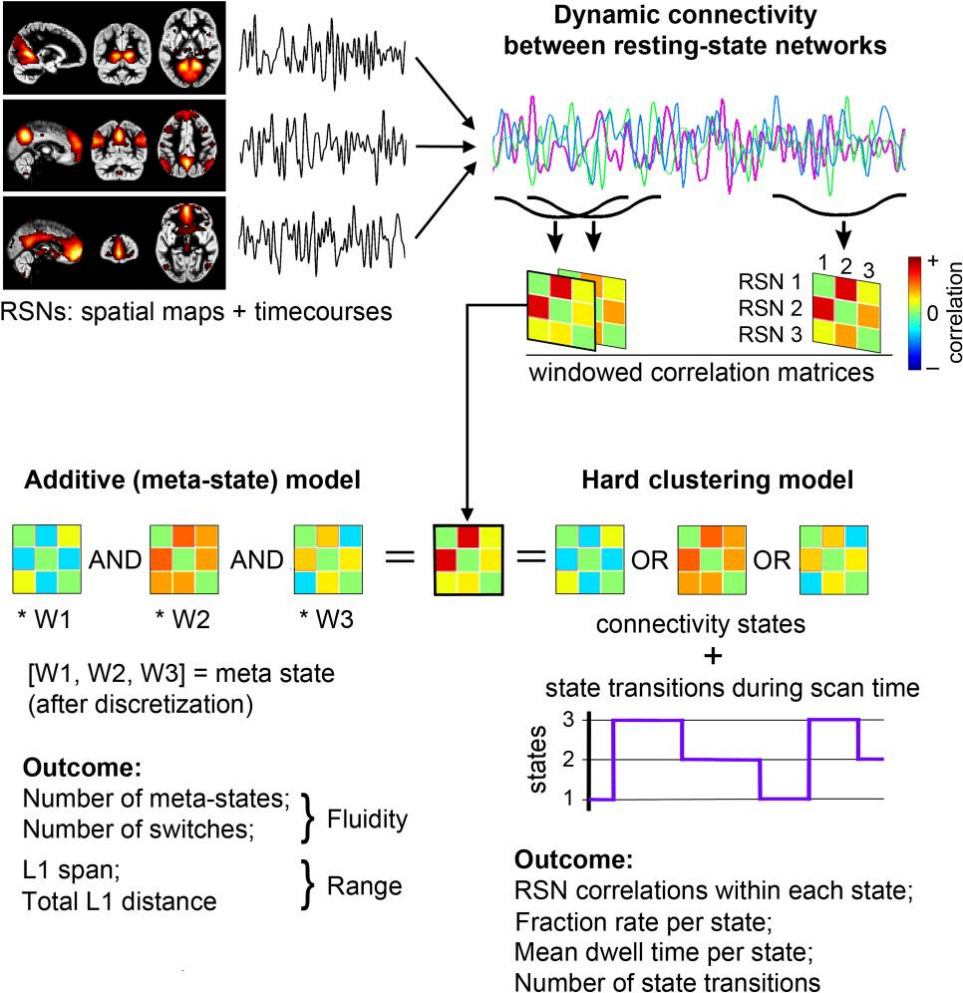
**A schematic illustration of the two dynamic FNC analyses; the hard clustering state and the meta-state connectivity analyses**.

The dynamic connectivity was examined in two ways, using both standard (hard clustering) and meta-state approaches, both incorporated in the Temporal dynamic FNC toolbox in GIFT.^6,7^ In both dynamic FNC analyses, covariance values were residualized with respect to log-transformed mean framewise displacement. Both analyses started from the extraction of windowed FNC matrices. Pairwise correlations between RSNs were computed using a sliding Gaussian-tapered (alpha value = 6 s) rectangular window with a length of 60 s in steps of one repetition time (2 s), yielding 198 windowed FNCs for each of 153 pair correlations per subject. The calculation of correlation estimates included five runs of L^1^ regularization within the graphical lasso framework.^35^ The windowed FNC matrices were fed into two dynamic FNC analyses as illustrated by Fig. 2.

#### Dynamic FNC estimated with hard clustering

In the analysis,^6^ each windowed FNCs was replaced with one of the prototype connectivity states that the windowed FNC resembled most closely. This made it possible to describe the connectivity dynamics as a series of transitions between these few reoccurring states. Before clustering, all the windowed FNC were concatenated along the subject × time dimension. The *k*-means clustering algorithm (iterated a maximum of 200 times), with the city distance measure, was used to define four stable FNC states (cluster centroids) based on a subset of subject windows corresponding to local maxima in the standard deviation. The number of the FNC states was equal to four as determined by the elbow criterion. The cluster centroids were used as starting points for clustering all of the data (98 subjects * 198 windows = 19 404 instances), resulting in the assignment of each windowed FNC to one of the four FNC states. Each state had to be present in a minimum of 10 windows for each subject.

Two-sample *t*-tests on group medians of correlation were performed for each pair correlation within each state. In addition, the percentage of scanning time spent in each state, i.e. the fraction rate, a mean time a subject was at a specific state, i.e. dwell time and the number of transitions between states was computed and compared between groups. To check the stability of the results, we repeated the hard clustering state FNC analysis for window widths of 50 and 44 s (see Supplementary Table 2A–C and Supplementary Fig. 2).

#### Dynamic FNC estimated with the meta-state method

In a meta-state analysis,^7^ each windowed FNC was replaced by a sum of prototype connectivity patterns obtained in a similar way as is described above for hard clustering method. The connectivity patterns were weighted according to their contribution to each windowed FNC. The weights were replaced with the signed quartile that the weight value falls into in the range {±1, 2, 3, 4}. Each discretized vector of connectivity pattern weights formed a meta-state. We created a vector of five distinct connectivity patterns, thus obtaining a five-dimensional characterization of each subject’s 153-dimensional (number of pair combinations for 18 independent component time courses) structure in each of the 198-time windows.

In contrast to the hard clustering method reducing all time-varying connectivity to a sequence of a few states, an additive model to describe each windowed FNC is used while having a vast variety of possible contributing meta-states. In this method, quantification of the temporal connectivity dynamics is characterized by four metrics: (i) the number of distinct meta-states the subject occupied during the scan time; (ii) the number of times the subjects switched from one meta-state to another; (iii) the largest L1 distance (span) between the occupied meta-states and (iv) the overall distance travelled by the subject through meta-state space (the sum of all L1 distances between successive meta-states). The first two metrics characterize fluidity, while the second two describe the dynamic range. Higher values for each of the metrics are associated with higher dynamism. Though we used five connectivity patterns as providing reliable characterization of the meta-state connectivity^7^ we additionally checked the stability of our results by repeating the analysis for four and six connectivity patterns while keeping the window width equal to 60 s (see Supplementary Table 3).

#### Statistical analyses

The group differences in head motion parameters during fMRI the Mann–Whitney U-test. When finding local maxima for spatial map of each RSN (see Supplementary Table 1), one sample *t*-test was used. In the hard clustering state analysis, two-tailed two-sample *t*-tests on group medians of pair correlations between RSNs were performed within each state with the threshold of *P* < 0.05 after false discovery rate (FDR) correction. The fraction rate, dwell time and number of transitions were compared between groups with a two-tailed two-sample *t*-test and significance threshold of *P* < 0.05, with FDR correction. The results of the meta-state analysis were compared between the groups within each metric using a two-tailed two-sample *t*-test, with a significance threshold of *P* < 0.05 and FDR correction.

## Results

### Subjects

The initial sample consisted of 35 adolescents born VPT and 35 controls, who were eligible after excluding cases with large susceptibility artefacts and anatomical abnormalities in the visual inspection of comprehensive MRI. As a result of the subject selection steps (see the Methods section for details), resting-state fMRI data from 24 adolescents born VPT (14 boys and 10 girls) and 32 controls (14 boys and 18 girls) were used in ICA. The groups did not differ in mean age (*P* = 0.4, two-sample *t*-test) or gender proportions (*P* = 0.42 in Fisher’s exact two-tail test). The mean age of all participants at the time of the resting-state fMRI scan was 12.9 (12.3 and 13.8). The perinatal characteristics of adolescents born VPT are shown in Table 1. The drop-out analysis, between the adolescents who had refused to participate either at this or earlier stages of this longitudinal cohort study, is also shown in Table 1. There were no significant differences between the background characteristics of participating or refusing adolescents born VPT.

**Table 1:** Characteristics of the adolescents born VPT participating the study (*n* = 24) compared with those who withdrew (*n* = 67)

	Participants, *n* = 24	Drop-outs, *n* = 67	*P*-value	Controls *n* = 32
Gestational age in weeks, mean (SD) [min, max]	29 + 1 (2 + 4) [24 + 5, 33 + 6]	29 + 5 (2 + 6) [23 + 0, 34 + 0]	0.4	40 + 0 (1 + 0) [37 + 0, 42 + 0]
Birth weight in grams, mean (SD) [min, max]	1201 (352) [620, 2120]	1258 (362) [565, 2025]	0.5	3578 (435) [2830, 4580]
Gender	14 boys and 10 girls	46 boys and 21 girls	0.4	15 boys and 17 girls
Age at scanning in years, mean (SD)	12.9 (0.5)^c^			12.8 (0.3)^c^
Small for gestational age, *n* (%)^a^	8 (33)	19 (28)	0.6	
Antenatal steroids, *n* (%)	23 (96)	60 (90)	0.7	
Caesarean section, *n* (%)	16 (67)	38 (57)	0.4	
Multiple birth, *n* (%)	9 (38)	18 (27)	0.3	
Necrotizing enterocolitis, *n* (%)	0/23 (0)	3/64 (5)	0.6	
Retinopathy of prematurity, laser-treated, *n* (%)	1/23 (4)	3/64 (5)	0.9	
Sepsis or meningitis, *n* (%)	1 (4)	5 (7)	1	
Bronchopulmonary dysplasia, *n* (%)	2 (8)	8 (12)	1	
Parental education ≤ 12 years, *n* (%)				
Fathers	12/22 (55)	17/59 (29)	0.5	
Mothers	8 (33)	28/59 (47)	0.7	
FSIQ^b^ at 11 years, median (SD)	92.0 (15.6)	89.0 (17.1)	0.06	
FSIQ <70, *n* (%)	2/23 (9)	8/50 (16)	0.5	

a Birthweight <2 SD compared to the normative growth charts.

b FSIQ measured with Wechsler Intelligence Scale for Children-IV.

c The age at scanning did not differ between the preterm and term-born adolescents (*P* = 0.4).

Three of the VPT adolescents had minor lesions (minimal frontal parenchymal lesions, prominent frontal horn and slightly widened lateral ventricles, respectively) in comprehensive MRI. None of the adolescents born VPT fulfilled the criteria of significant cognitive impairment, even though two boys had FSIQ <70. Neither of these two had visible lesions in comprehensive MRI. One of the adolescents was born VPT and one of the controls had an attention deficit hyperactivity disorder diagnosis and medication. All the findings remained statistically significant after excluding adolescents with a FSIQ <70 or with attention deficit hyperactivity disorder.

### Group difference by the hard clustering dynamic FNC analysis

**Figure 3:**
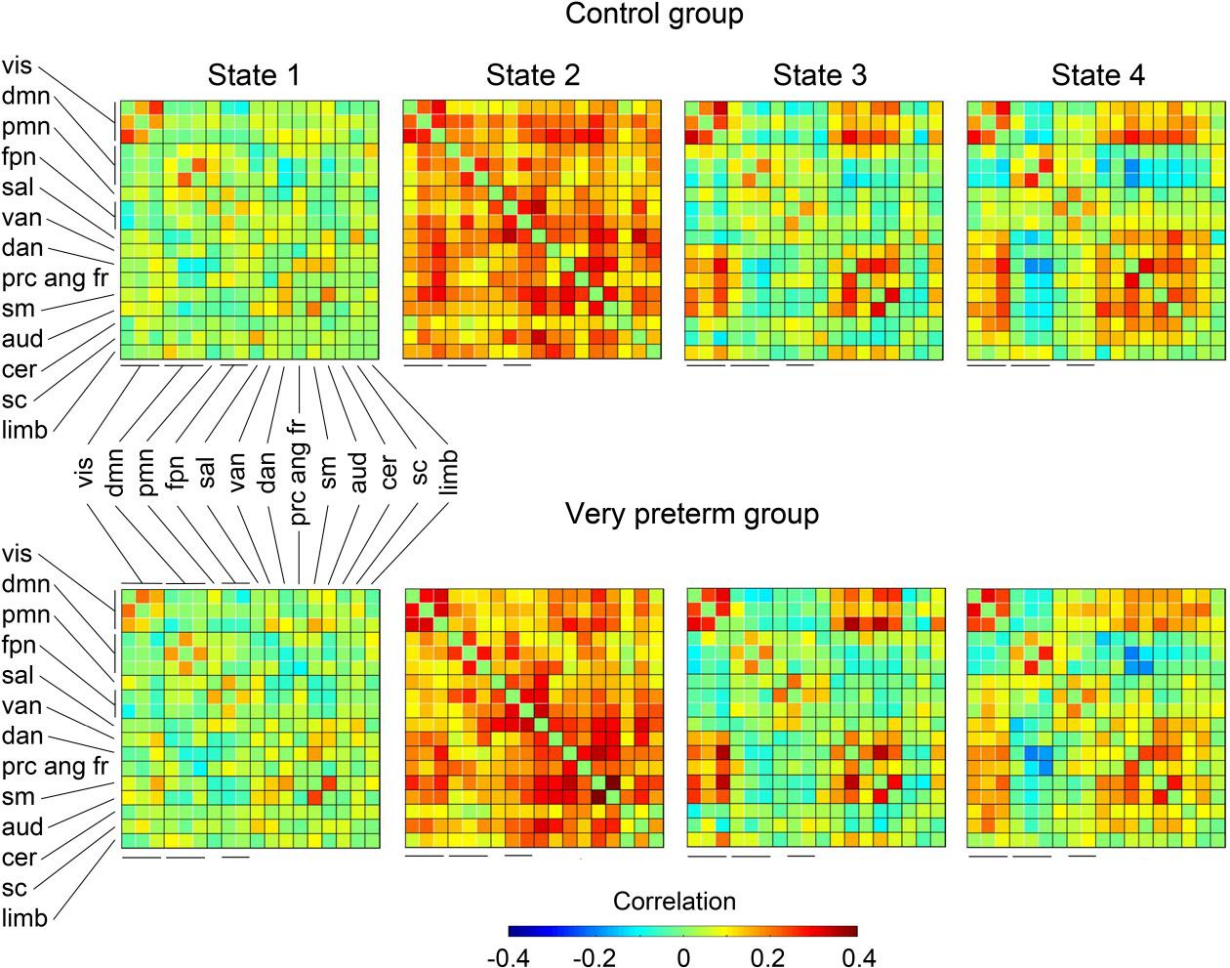
**Correlation matrices representing the four connectivity states of the hard clustering state FNC analysis for both groups**. State 1 shows the weakest, State 2 the strongest and States 3 and 4 the intermediate strength connectivity.

**Figure 4:**
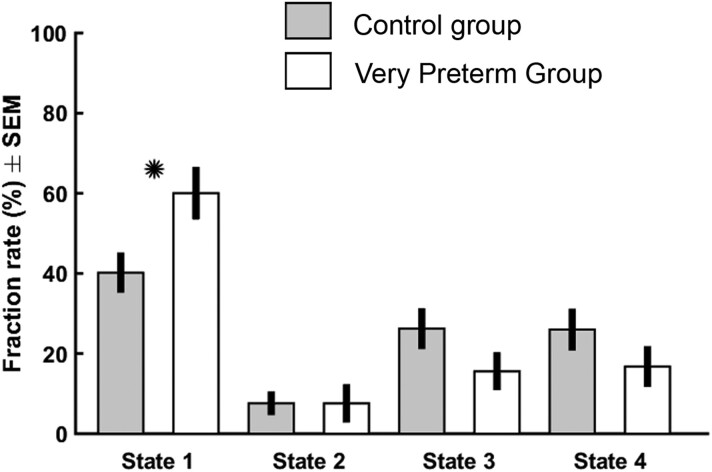
**The fraction rates of the adolescents born VPT and term-born controls.** The significant difference in the fraction rate in the least active state is highlighted with *. The groups were compared using a two-sample *t*-test, and the significance threshold was set at *P* < 0.05 with FDR correction.

Correlation matrices representing four connectivity states for both groups are displayed in Fig. 3. State 1 showed the weakest, State 2 the strongest and States 3 and 4 the intermediate connectivity. No pairwise differences were observed in any of the hard clustering state FNC states between the two study groups. The groups differed (*P* = 0.02, FDR-corrected) in the fraction rate in the least connected state, State 1. The adolescents born VPT had a higher fraction rate (mean 60.0%, 95% confidence interval 48.3–71.8) than controls (mean 40.2%, 95% confidence interval 31.7–48.6) as shown in Table 2, Fig. 4 and detailed in Supplementary Table 2A–C. In additional analyses, the difference was confirmed when window width was set at 50 s. The mean dwell time and a number of state transitions were not significantly different in any state between the groups no matter the window width (Supplementary Table 2A–C).

**Table 2:** Mean state wise fraction rates as percentages, dwell times and transition frequencies, their standard errors of means (SEM) and 95% confidence intervals (95% CIs) for both adolescents born VPT and full term-born controls (controls), and the FDR corrected *P*-values of the comparisons with two sample *t*-test

Fraction rate %	VPT Mean (SEM) [95% CI]	Controls Mean (SEM) [95% CI]	*P*-values
State 1	60.0 (5.69) [49.6–70.4]	40.2 (4.14) [32.2–49.2]	0.02
State 2	7.60 (3.90) [1.34–13.9]	7.59 (2.07) [2.17–13.0]	1.0
State 3	15.6 (3.88) [6.63–24.3]	26.2 (4.21) [18.5–34.0]	0.2
State 4	16.8 (4.20) [7.37–26.2]	26.0 (4.35) [17.8–34.1]	0.2

Dwell time	VPT Mean (SEM) [95% CI]	Controls Mean (SEM) [95% CI]	*P*-values
State 1	59.4 (12.3) [41.6–77.2]	30.3 (4.35) [14.9–45.7]	0.07
State 2	8.20 (3.87) [2.00–14.4]	9.71 (2.05) [4.34–15.1]	0.7
State 3	12.3 (2.27) [3.18–21.4]	23.0 (4.91) [15.1–30.9]	0.2
State 4	13.3 (2.81) [6.86–19.8]	19.2 (3.02) [13.6–24.8]	0.2

Number of transitions	VPT Mean (SEM) [95% CI]	Controls Mean (SEM) [95% CI]	*P*-values
	5.88 (0.72) [4.44–7.32]	7.13 (0.62) [5.87–8.37]	0.9

### Group difference by the meta-state dynamic FNC analysis

**Figure 5:**
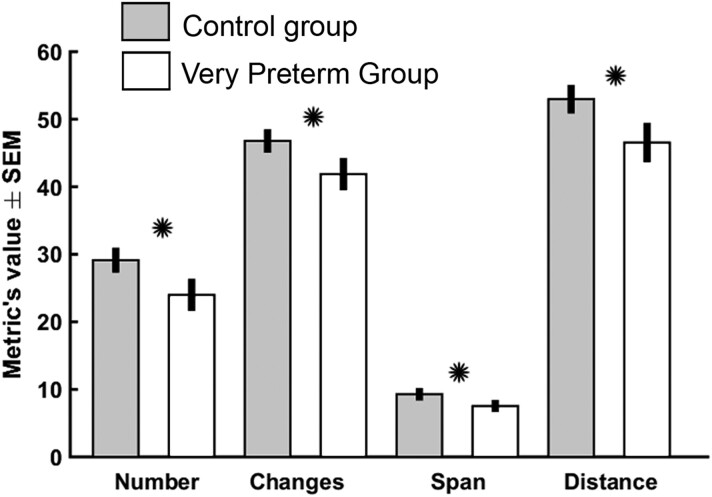
**The results of the four meta-state FNC metrics in adolescents born VPT and controls**. Significant differences are highlighted with *. The groups were compared using a two-sample *t*-test, and the significance threshold was set at *P* < 0.05 with FDR correction.

All of the meta-state FNC metrics—i.e. the number of states, changes between the states, state span and total distance—demonstrated significant (*P* < 0.05, FDR-corrected) reduction in the VPT group when compared to controls. The numeric results of the meta-state FNC analysis are shown in Table 3 and presented in Fig. 5. The additional analysis confirmed a significant group difference for span for all meta-state dimensions (4, 5 or 6 connectivity patterns), and the number of changes when using six connectivity patterns (Supplementary Table 3).

**Table 3:** Mean values of the meta-state FNC analyses and their standard errors of means (SEM) and 95% confidence intervals (95% CIs) for adolescents born VPT and term-born controls (controls), and the FDR corrected *P*-values of the comparisons with two sample *t*-tests

Meta states	VPT Mean (SEM) [95% CI]	Controls Mean (SEM) [95% CI]	*P*-values
Count	24.0 (1.86) [20.6–26.4]	29.1 (1.33) [26.2–32.0]	0.03
Count of changes	41.9 (1.87) [38.6–45.1]	46.8 (1.20) [44.0–49.6]	0.03
Mean span	7.54 (0.37) [6.70–8.39]	9.28 (0.39) [8.54–10.0]	0.01
Overall distance	46.5 (2.39) [42.4–50.7]	53.0 (1.59) [49.3–56.6]	0.03

## Discussion

We examined dynamic FNC in 13-year-old adolescents born VPT and their term-born controls. Opposite to our hypothesis, there were no group differences in pairwise connectivity, as revealed by the hard clustering dynamic FNC analysis. However, the adolescents born VPT stayed in the least active connectivity state for a higher fraction of the total scanning time. Though a clear interpretation of the functional meaning for a certain connectivity state obtained with hard clustering method seems difficult, a higher dimensional meta-state analysis clearly showed diminished fluidity and range in connectivity for the VPT group. Thus, the VPT group occupied fewer meta-states of the lower dynamic range, switched between them less often and showed a shorter overall distance through the meta-state space compared to controls.

This is, to our knowledge, the first study assessing dynamic FNC in adolescents born VPT, though static FNC analysis has been used by others for the prematurely born. Two static FNC studies by Degnan *et al*.^12,13^ with typically functioning children born late preterm (gestational age 34–36), showed similar spatial orientation, but a functional hyperconnectivity in the default mode network, central executive network and salience network; and an increased anti-correlation within the central executive network at the age of 9–13 compared to controls. The study populations in their studies consisted of a combined sample of children and adolescents born late preterm who were also low to moderate-income twins. Both the population and the methodology make these studies not directly comparable with our study.

In a longitudinal static FNC study Rowlands *et al*.^14^ found that at 8 years of age (13 preterm and 12 term-born children), the resting-state static FNC was similar between the groups. There was a difference between these groups at the age of 16, and the rsfMRI connectivity was markedly increased in the preterm group in several areas including occipito-temporal cortex, inferior frontal gyri and orbitofrontal and anterior prefrontal cortices. It remains unclear whether the present cohort would acquire similar changes in the static FNC connectivity patterns later in adolescence. The study of Rowlands *et al*.^14^ also included late preterm-born infants, which makes the study not directly comparable with our study.

One more static FNC study revealed both increased and decreased connectivity when comparing adolescents born VPT to the controls.^15^ Differences were found especially in the networks involved in higher-order cognitive functions. The adolescents born VPT had a similar mean age compared to ours, but the age range was 10–16. This complicates the comparison with our study, as well as a seed-based analysis technique used by the authors. Also, a study by Wilke *et al*.^11^ found a group difference between adolescents born VPT and controls in language-related networks in static FNC. Their VPT and control groups differed from each other in terms of the age at scan, which might explain the between-group differences they found.

In this study, we did not consider the static FNC because of the contemporary conclusion of the dynamic fluctuation of brain connectivity at rest. The differences between adolescents born VPT and controls were found in the timing of hard clustering state FNC and meta-state FNC. These methods are more fine-grained and sensitive than static FNC analysis. We also used ICA, which allowed us to perform the analysis without a priori hypothesis. Previously, Hutchinson and Morton^36^ found differences in the timing of hard clustering state FNC and meta-state FNC despite the lack of differences in the correlation matrices of hard clustering state FNC. They found an age-related decrease in the dwell time and transition frequency in a population with an age range of 9–32 years (32 children and 19 adults). This highlights the possibility of differences in temporal dimensions without differences in the group connectivity matrices.

Regarding our meta-state analysis, we found that children born VPT occupied fewer states during the scan, made fewer switches between meta-states and travelled a shorter distance in the meta-state space. This means that the fluidity and range of RSN connectivity in the preterm-born group are decreased.

Previous studies have shown a combined type of attention deficit hyperactivity disorder to associate with increased meta-state span in children and adolescents which was also found in our study.^18^ The adolescents born VPT usually have traits belonging to the inattentive subtype.^37^

It is also notable that the association between prematurity and temporal differences in resting-state FNC discovered in this study persisted when excluding adolescents with attention deficit hyperactivity disorder. This might suggest that our results were not driven by specifically attention deficit hyperactivity disorder-related alterations in FNC, but by the prematurity itself. On the other hand, the slow wave power in EEG at rest has previously been shown to be similarly increased in both adults with attention deficit hyperactivity disorder born full term and adults born VPT without attention deficit hyperactivity disorder. This supports the hypothesis of similarities in neural functioning between these groups.^38^

Changes in hard clustering state dynamic FNC and meta-state dynamic FNC metrics have previously been observed in autism spectrum disorder, but also children with milder adversities in social competence.^39^ Rashid *et al*.^39^ found a longer dwell time in the least connected state in children with an autism spectrum disorder. De Lacy *et al*.^40^ found a significant decrease in the number of meta-state changes in adults with an autism spectrum disorder and Fu *et al*.^19^ in all meta-state metrics in adolescents with autism spectrum disorder. We have previously reported a trend of poorer social competence in adolescents born VPT.^25^

### Limitations and technical discussion

The lack of group differences in pair-wise RSN comparisons obtained with hard clustering method could suggest that there is only a negligible difference in connectivity patterns between the groups. Additionally, the moderate sample size could influence the detectability of such differences. Moreover, most of the participants excluded due to head motion were in the VPT group, which could diminish the differences.

Our series of 228 time points (7.6 min) exceeds 6 min duration that should provide a stable connectivity signal.^41^ We were interested in examining large-scale connectivity, so we used a relatively low-order ICA model and analysed only the connectivity between, not within RSNs. Finally, the differences in preprocessing and certain ICA and FNC parameters may lead to somewhat different results between studies.

### Future prospective

Our study continues the discussion on functional disruptions in the neural networks of adolescents born VPT. Understanding the disruptions in brain functioning caused by VPT birth is important to ensure the proper growth environment and adequate support in daily life. We highlight the possible adverse effects in adaptive brain functions, in terms of dynamic fluidity, related to VPT birth.

## Conclusion

The long-term outcomes of VPT infants have improved over the last decades, but adolescents born preterm still suffer from anxiety, social problems and inattentiveness. In this study, we found aberrations in several characteristics of dynamic functional connectivity in adolescents born preterm. These findings resemble those made in previous studies on combined-subtype attention deficit hyperactivity disorder and autism spectrum disorder, which are the extreme versions of the features typical of the preterm behavioural phenotype. These findings could capture the brain mechanisms underlying the clinically observed behavioural features and the effects of prematurity on brain function.

## Supplementary Material

fcad009_Supplementary_Data

## Data Availability

The data are only to be made available via a request to the Authors because of the clinical nature of the data sample and ethics regarding this. The possible requests are individually inspected, and the availability and agreements are carefully considered to meet the ethical and privacy policy of the University of Turku and Turku University Central Hospital. The data were processed utilizing standard codes of free and open-source software.

## Abbreviations

BOLD: blood oxygen level dependent
fMRI: functional MRI
FNC: functional network connectivity
ICA: independent component analysis
RSN: resting-state network
