# Supplementary material for: Altered temporal connectivity and reduced meta-state dynamism in adolescents born very preterm
Source: Brain Commun. 2023 Feb 7;5(1):fcad009. doi: 10.1093/braincomms/fcad009 (PMC9927875; doi:10.1093/braincomms/fcad009)
Supplement: fcad009_Supplementary_Data [file fcad009_supplementary_data.pdf]

Supplementary materials

Resting state networks

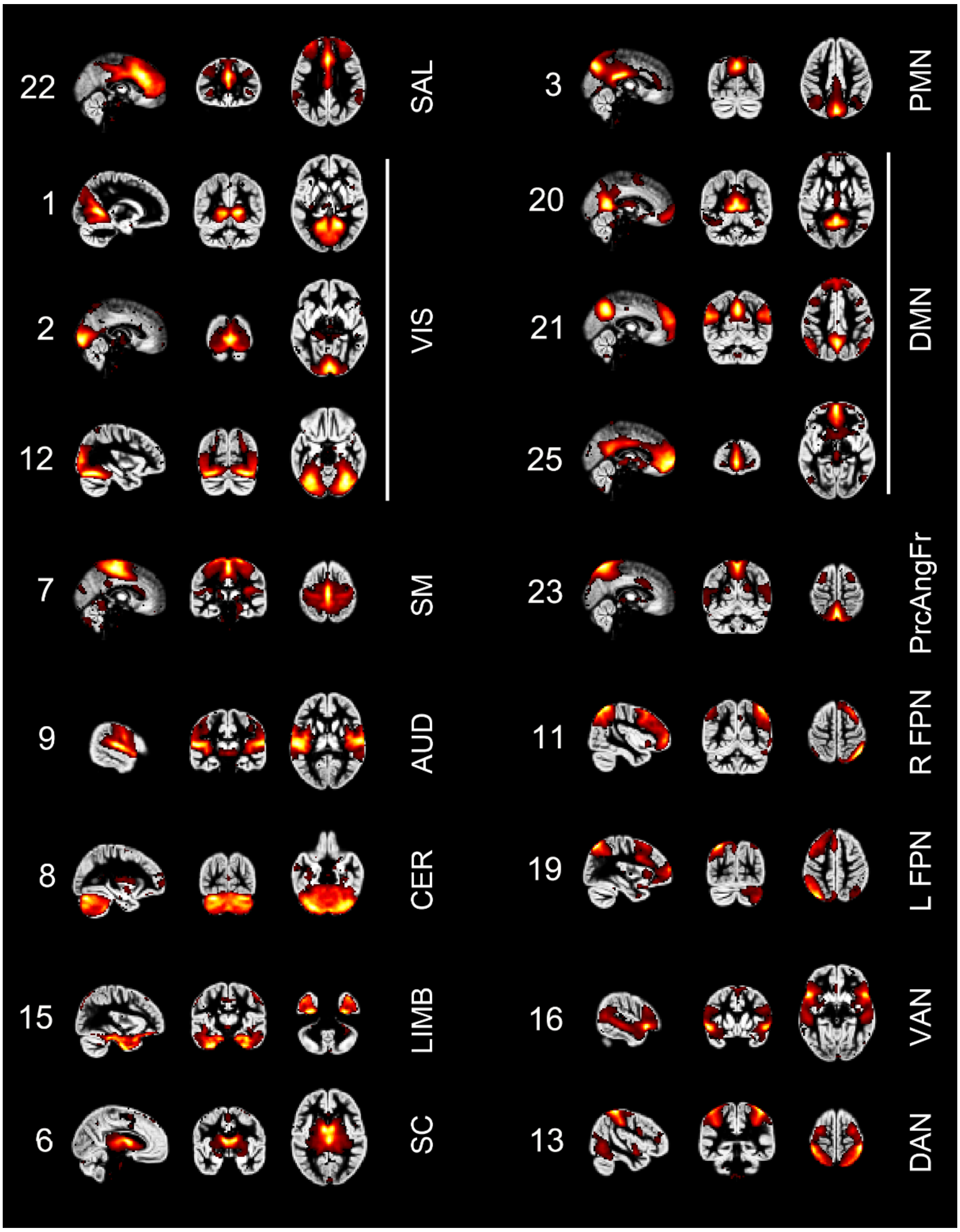

Supplementary Figure 1. Numbered spatial maps of independent components identified as resting state networks belonging to the following domains: Default Mode Network (DMN), Salience Network (SAL), Posterior Memory Network (PMN), Left and Right Fronto-Parietal Networks (L FPN and R FPN), Ventral Attention Network (VAN), Dorsal Attention Network (DAN), Network with maxima in superior precuneus, angular and frontal cortices (PrcAngFr), Visual Network (VIS), Sensory-Motor Network (SM), Auditory Network (AUD), Subcortical Network (SC), Cerebellar Network (CER), and Limbic Network (LIMB). Three orthogonal sections for each spatial map above t-value = 3 in one-sample t-test are shown. The numbers provide correspondence with Supplementary Table 1.

Supplementary Table 1. The location of the main maxima (bold font) and submaxima (regular font) of the independent components (ICs), sorted according to domains of resting state networks (RSNs). The values of x, y, and z are coordinates in the Montreal Neurological Institute brain space.

RSN domain	IC number	Brain region	t-value	x	y	z
VIS	**1**	R Calcarine	**27.65**	**21**	**-60**	**6**
		Cuneus	27.38	0	-81	30
		R Calcarine	25.38	3	-69	12
	**2**	Calcarine	**26.53**	**0**	**-90**	**-3**
		L Calcarine	25.84	-9	-90	0
		Lingual	21.54	0	-78	3
	**12**	R Middle Occipital	**22.39**	**33**	**-90**	**6**
		L Middle Occipital	20.39	-24	-93	12
		R Fusiform	20.28	24	-75	-9
DMN	**20**	L Medial Superior Frontal	**30.01**	**-3**	**60**	**9**
		L Ventral Medial Frontal	**14.65**	**-3**	**54**	**-9**
		R Angular gyrus	**11.82**	**48**	**-69**	**33**
		R Superior Frontal	**11.46**	**24**	**33**	**45**
		L Middle Occipital / Angular	**10.91**	**-42**	**-75**	**33**
		L Middle Frontal	**10.69**	**-24**	**27**	**51**
	**21**	L Precuneus	**23.84**	**-9**	**-54**	**36**
		R Medial Superior Frontal	**18.73**	**3**	**51**	**30**
		R Angular Gyrus	**18.29**	**51**	**-63**	**33**
		L Angular Gyrus	**17.55**	**-51**	**-57**	**27**
		R Middle Temporal gyrus	**14.53**	**60**	**-12**	**-18**
	**25**	Anterior Medial Frontal	**24.52**	**0**	**57**	**0**
		L Posterior Cingulate	**18.26**	**-3**	**-48**	**30**
PMN	**3**	L Posterior Cingulate	**29.43**	**-3**	**-42**	**21**
		Precuneus	29.04	0	-78	42
		L Inferior Parietal	**12.12**	**-39**	**-57**	**48**
		R Angular / Inferior Parietal	**9.93**	**42**	**-63**	**48**
FPN R	**11**	R Angular / Inferior Parietal	**21.55**	**51**	**-60**	**45**
		R Inferior / Middle Frontal	**17.76**	**42**	**48**	**-3**
		L Cerebellum	**17.24**	**-12**	**-87**	**-27**
		R Middle Temporal	**12.76**	**60**	**-39**	**-6**

		L Inferior Parietal	**11.77**	**-51**	**-54**	**51**
FPN L	**19**	L Inferior / Middle Frontal	**21.01**	**-48**	**27**	**21**
		L Superior Frontal	**15.96**	**-36**	**-69**	**54**
		R Cerebellum	**13.25**	**15**	**-84**	**-27**
		L Middle Temporal	**11.89**	**-57**	**-45**	**-9**
SAL	**22**	L Anterior Cingulate	**25.23**	**-3**	**18**	**36**
		L Insula	**18.62**	**-42**	**12**	**-6**
		R Superior Temporal	**14.41**	**51**	**15**	**-9**
VAN	**16**	R Insular / Superior Temporal	**21.25**	**51**	**12**	**-6**
		R Inferior Frontal	14.94	51	24	-6
		R Middle Temporal	14.32	57	-39	6
		L Inferior Frontal	**17.71**	**-54**	**18**	**-3**
		L Inferior Frontal	17.43	-45	24	-6
		L Middle Temporal	16.14	-54	-36	0
DAN	**13**	L Inferior Parietal	**22.92**	**-42**	**-36**	**45**
		R Inferior Parietal	**18.43**	**36**	**-39**	**51**
		L Superior Frontal	**14.43**	**-24**	**-9**	**57**
		L Inferior Occipital / Temporal	**14.18**	**-48**	**-69**	**-3**
		L Precentral / Inferior Frontal	**13.95**	**-54**	**6**	**30**
		R Inferior Temporal	**12.51**	**54**	**-60**	**-9**
		R Precentral / Inferior Frontal	**11.04**	**57**	**9**	**30**
PrcAngFr	**23**	Precuneus	**23.92**	**0**	**-60**	**60**
		L Middle / Superior Frontal	**12.24**	**-24**	**6**	**60**
		R Middle / Superior Frontal	**10.19**	**24**	**6**	**60**
SM	**7**	Paracentral Lobule	**24.78**	**0**	**-27**	**66**
AUD	**9**	L Superior Temporal / Postcentral	**20.99**	**-60**	**-18**	**15**
		L Superior Temporal	**20.24**	**60**	**-6**	**9**
CER	**8**	R Cerebellum	**22.15**	**18**	**-81**	**-33**
		L Cerebellum	20.1	-12	-72	-27
SC	**6**	R Thalamus	**25.66**	**3**	**-24**	**12**
		L Caudate	25.25	-12	-3	15
		R Caudate	23.75	12	-3	15
		L Caudate	19.09	-9	6	12
LIMB	**15**	L Parahippocampal / Amygdala	**14.41**	**-18**	**-6**	**-27**
		L Fusiform	11.22	-36	-39	-24

	L Temporal Pole	9.47	-45	12	-36
	L Inferior Frontal	10.24	-39	27	-18
	L Inferior Frontal	9.19	-27	27	-21
	R Temporal Pole	**11.85**	**51**	**9**	**-30**
	R Inferior Frontal	11.38	30	24	-21
	R Parahippocampal / Amygdala	10.35	18	-9	-24
	R Inferior Temporal / Fusiform	10.21	45	-39	-21
	R Inferior Frontal	9.87	39	30	-18

Additional dynamic functional network connectivity (FNC) analyses with different window sizes and meta-state space dimensions

The choice of window length is a trade-off decision. A short window would result in a smaller number of data points (volumes) and an increased risk of spurious correlations, while a long window could impede the detection of temporal variations of interest.[1] In theory, a recommended minimum window length equal to $1/f_{min}$ optimally avoids the detection of spurious correlations.[2] This would suggest 100 s for our study with $f_{min} = 0.01$ Hz. Such a wide window, however, would not allow us to detect dynamic FNC at faster, blood oxygen level dependent signal frequencies that are of interest. Zalesky and Breakspear (2015)[3] demonstrated that, in theory, a much shorter window (e.g., 40 s) could still be used in hard clustering state FNC methods while maintaining a reasonable control of false positives. In practice, window length in the range of 30–60 s seems to be acceptable for achieving reliable dynamic FNC results.[4–6]

We have chosen a 60 s window length for our main analysis[7-8]. However, we performed additional validation analyses using window lengths of 50 s and 44 s[9–10]. The estimation of the number of states with the elbow criterion resulted in four stable states for all three window lengths that we used for conventional hard clustering state FNC analysis.

All of the hard clustering state FNC analyses yielded a very similar connectivity matrices within each state despite different window lengths, as illustrated by Supplementary Figure 2. Results of the statistical analysis for fraction rate, dwell time, and number of transitions are listed in Supplementary Tables 2A, 2B, and 2C, respectively.

For meta-state connectivity, we tested the stability of results obtained in our main analysis with connectivity patterns = 5 by also testing variants with 4 and 6 connectivity patterns while keeping window length equal to 60 s. Results of the statistical analysis of meta-state metrics are listed in Supplementary Table 3.

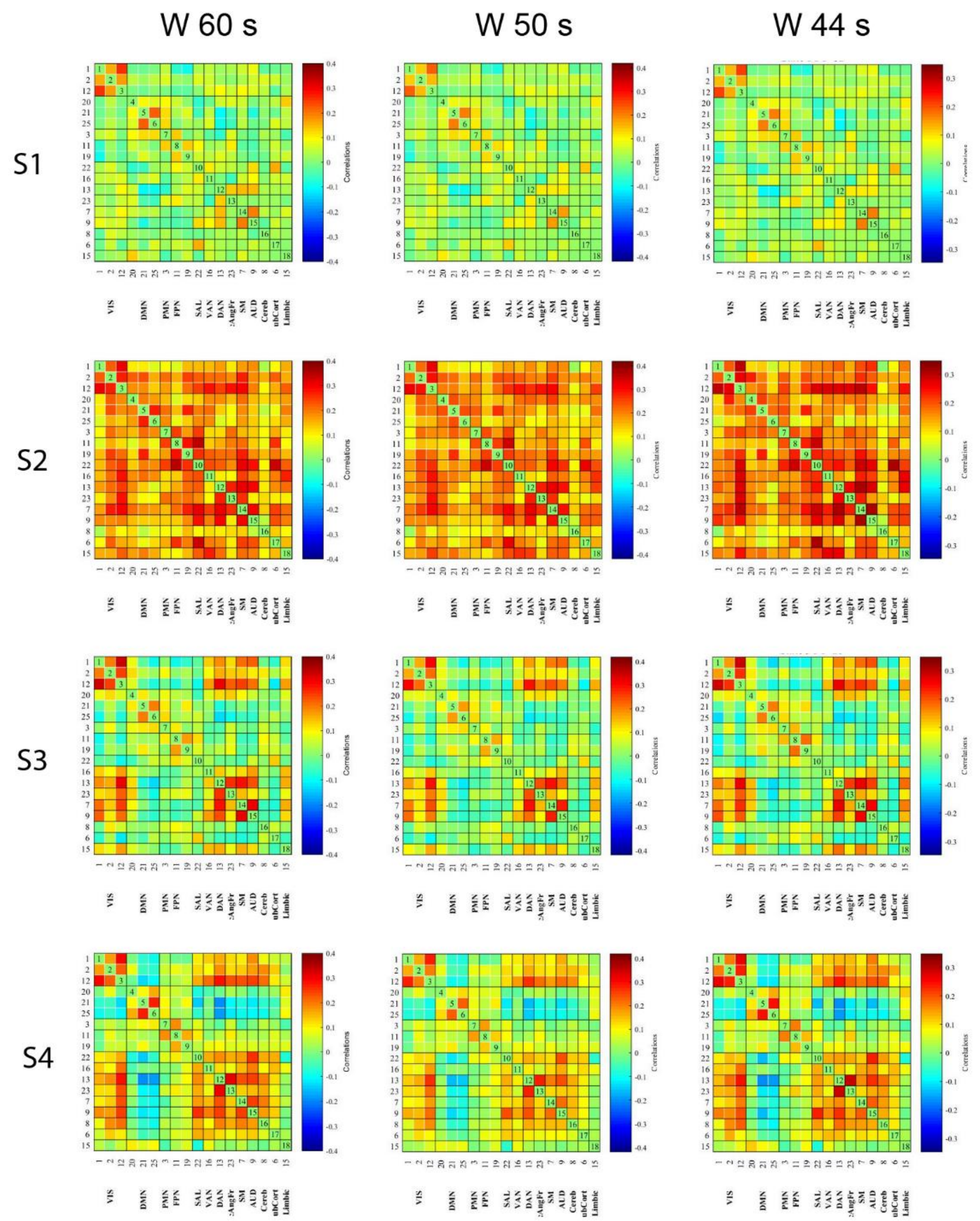

Supplementary Figure 2. Correlation matrices of four connectivity states (S1–S4) obtained in three hard clustering dynamic FNC analyses using window widths (W) of 60, 50, and 44 s for the control group. Note a remarkable similarity of the connectivity patterns across the window widths.

Supplementary Table 2 A. Fraction rate: group means with standard error of the mean (SEM) and 95% confidence interval (95% CI: lower and upper limits), as well as group differences (two sample t-test) as obtained by the hard clustering dynamic FNC analyses with different window widths (60, 50, and 44

seconds). Negative t-values denote the “very preterm group > control group” differences. The p-values were adjusted for FDR. Significant ($p<0.05$) differences are marked with asterisks.

Window width, s		State 1	State 2	State 3	State 4
	**Control group**				
	Mean	40.20	7.59	26.23	25.98
	SEM	4.14	2.07	4.21	4.35
	95% CI	31.2, 49.2	2.17, 13.0	18.47, 34.0	17.84, 34.12
	**Preterm group**				
**60**	Mean	60.04	7.60	15.59	16.77
	SEM	5.69	3.90	3.88	4.20
	95% CI	49.6, 70.4	1.34, 13.86	6.63, 24.26	7.37, 26.18
	**Difference**				
	T-value	-2.89	-0.001	1.80	1.48
	P-value	0.022*	0.999	0.155	0.192
	**Control group**				
	Mean	40.99	6.93	25.75	26.32
	SEM	3.71	1.86	3.80	3.83
	95% CI	32.68, 49.30	1.85, 12.0	18.70, 32.80	19.33, 33.32
	**Preterm group**				
**50**	Mean	60.71	6.51	15.44	17.34
	SEM	5.39	3.72	3.57	3.43
	95% CI	51.12, 70.31	0.65, 12.37	7.30, 23.58	9.26, 25.42
	**Difference**				
	T-value	-3.11	0.11	1.92	1.68
	P-value	0.012*	0.914	0.120	0.130
	**Control group**				
	Mean	43.81	7.71	23.86	24.62
	SEM	3.83	1.80	3.60	3.58
	95% CI	35.36, 52.26	2.56, 12.86	17.24, 30.49	17.80, 31.43
	**Preterm group**				
**44**	Mean	58.20	7.67	15.09	19.05
	SEM	5.40	3.84	3.31	3.62
	95% CI	48.43, 67.95	1.72, 13.61	7.44, 22.74	11.18, 26.93
	**Difference**				
	T-value	-2.23	0.01	1.74	1.07
	P-value	0.119	0.992	0.176	0.385

Supplementary Table 2 B. Dwell time: group means with standard error of the mean (SEM) and 95% confidence interval (95% CI: lower and upper limits), as well as group differences (two sample t-test) as obtained by the hard clustering dynamic functional network connectivity analyses with different window widths (60, 50, and 44 seconds). Negative t-values denote the "very preterm group > control group" differences. The p-values were adjusted for FDR. No significant ($p<0.05$) differences were found.

Window width, s		State 1	State 2	State 3	State 4
	**Control group**				
	Mean	30.28	9.71	23.03	19.20
	SEM	4.35	2.05	4.91	3.02
	95% CI	14.86, 45.70	4.34, 15.09	15.14, 30.93	13.60, 24.76
	**Preterm group**				
**60**	Mean	59.44	8.20	12.30	13.30
	SEM	12.29	3.87	2.27	2.81
	95% CI	41.63, 77.24	2.00, 14.41	3.18, 21.41	6.86, 19.75
	**Difference**				
	T-value	-2.48	0.37	1.78	1.38
	P-value	0.065	0.714	0.160	0.230
	**Control group**				
	Mean	25.46	8.87	21.19	17.81
	SEM	3.05	1.85	4.75	2.41
	95% CI	11.99, 38.94	3.68, 14.06	13.65, 28.73	13.61, 22.00
	**Preterm group**				
**50**	Mean	50.82	7.75	11.74	10.75
	SEM	11.17	3.85	1.94	1.81
	95% CI	35.26, 66.39	1.76, 13.74	3.04, 20.44	5.91, 15.59
	**Difference**				
	T-value	-2.47	0.28	1.65	2.21
	P-value	0.063	0.778	0.140	0.063
	**Control group**				
	Mean	24.64	9.13	14.88	14.50
	SEM	3.10	1.65	1.94	1.65
**44**	95% CI	13.50, 35.78	4.15, 14.12	11.25, 18.50	11.48, 17.54
	**Preterm group**				
	Mean	39.35	7.24	10.75	10.55
	SEM	8.91	3.80	1.87	1.51

	95% CI	26.48, 52,21	1.48, 13.00	6.57, 14.94	7.05, 14.05
	**Difference**				
	T-value	-1,73	0.50	1.49	1.71
	P-value	0.185	0.621	0.188	0.185

Supplementary Table 2 C. Number of transitions: group means with standard error of the mean (SEM) and 95% confidence interval (95% CI: lower and upper limits), as well as group differences (two sample t-test) as obtained by the hard clustering dynamic FNC analyses with different window widths (60, 50, and 44 seconds). Negative t-values denote the "very preterm group > control group" differences. The uncorrected p-values are reported. No significant (p<0.05) differences were found.

**Window width, s**	**60**	**50**	**44**
**Control group**			
Mean	7.13	8.75	9.84
SEM	0.62	0.65	0.69
(95% CI)	5.87, 8.37	7.45, 10.05	8.45, 11.24
**Preterm group**			
Mean	5.88	7.46	9.5
SEM	0.72	0.75	0.80
(95% CI)	4.44, 7.32	5.95, 8.96	7.89, 11.10
**Difference**			
T-value	1.32	1.30	0.32
P-value	0.90	0.90	0.62

Supplementary Table 3. Meta-state metrics: group means with standard error of the mean (SEM) and 95% confidence interval (95% CI: lower and upper limits), as well as group differences (two sample t-test) for a window length of 60 s while using 4, 5, and 6 connectivity patterns. The p-values were adjusted for FDR. Significant (p<0.05) differences are marked with asterisks.

Number of connectivity patterns		Number of meta-states	Number of changes	Span	Total distance
**4**	**Control group**				
	Mean	16.00	34.72	7.40	38.31
	SEM	0.77	1.13	0.40	1.40
	95% CI	14.29, 17.71	32.32, 37.12	6.66, 8.15	35.45, 41.14
	**Preterm group**				
	Mean	14.46	30.88	5.96	33.29
	SEM	1.11	1.48	0.40	1.63
	95% CI	12.48, 16.43	28.10, 33.65	5.10, 6.82	30.03, 36.55
	**Difference**				
	T-value	1.18	2.10	2.56	2.33
	P-value	0.242	0.054	0.047*	0.047*
**5**	**Control group**				
	Mean	29.13	46.78	9.28	52.97
	SEM	1.33	1.20	0.39	1.59
	95% CI	26.21, 32.04	43.99, 49.58	8.54, 10.01	
	**Preterm group**				
	Mean	24.00	41.88	7.54	46.54
	SEM	1.86	1.87	0.37	2.39
	95% CI	20.63, 26.37	38.65, 45.10	6.70, 8.39	49.34, 56.59
	**Difference**				
	T-value	2.31	2.30	3.12	2.33
	P-value	0.025*	0.025*	0.012*	0.025*
**6**	**Control group**				
	Mean	40.16	56.19	11.72	66.75
	SEM	1.88	1.80	0.49	2.37
	95% CI	36.08, 44.23	52.21, 60.17	10.79, 8.09	61.61, 71.89
	**Preterm group**				
	Mean	34.58	48.79	9.17	56.83
	SEM	2.57	2.55	0.50	3.23
	95% CI	29.88, 39.29	44.20, 53.39	8.09, 10.24	50.90, 62.77
	**Difference**				
	T-value	1.795	2.44	3.60	2.53
	P-value	0.078	0.024*	0.003*	0.024*

References

1. Preti MG, Bolton TA, Van De Ville D. The dynamic functional connectome: State-of-the-art and perspectives. *Neuroimage*. 2017;160(December 2016):41-54. doi:10.1016/j.neuroimage.2016.12.061

2. Leonardi N, Van De Ville D. On spurious and real fluctuations of dynamic functional connectivity during rest. *Neuroimage*. 2015;104:430-436. doi:10.1016/j.neuroimage.2014.09.007

3. Zalesky A, Breakspear M. Towards a statistical test for functional connectivity dynamics. *Neuroimage*. 2015;114:466-470. doi:10.1016/j.neuroimage.2015.03.047

4. Shirer WR, Ryali S, Rykhlevskaia E, Menon V, Greicius MD. Decoding Subject-Driven Cognitive States with Whole-Brain Connectivity Patterns. *Cereb Cortex*. 2012;22(1):158-165. doi:10.1093/cercor/bhr099

5. Jones DT, Vemuri P, Murphy MC, et al. Non-Stationarity in the "Resting Brain's" Modular Architecture. He Y, ed. *PLoS One*. 2012;7(6):e39731. doi:10.1371/journal.pone.0039731

6. Allen EA, Damaraju E, Plis SM, Erhardt EB, Eichele T, Calhoun VD. Tracking Whole-Brain Connectivity Dynamics in the Resting State. *Cereb Cortex*. 2014;24(3):663-676. doi:10.1093/cercor/bhs352

7. Abrol A, Damaraju E, Miller RL, et al. Replicability of time-varying connectivity patterns in large resting state fMRI samples. *Neuroimage*. 2017;163:160-176. doi:10.1016/j.neuroimage.2017.09.020

8. Guo H, Liu L, Chen J, Xu Y, Jie X. Alzheimer classification using a minimum spanning tree of high-order functional network on fMRI dataset. *Front Neurosci*. 2017;11(DEC). doi:10.3389/fnins.2017.00639

9. Hindriks R, Adhikari MH, Murayama Y, et al. Can sliding-window correlations reveal dynamic functional connectivity in resting-state fMRI? *Neuroimage*. 2016;127:242-256. doi:10.1016/j.neuroimage.2015.11.055

10. Miller RL, Yaesoubi M, Turner JA, et al. Higher Dimensional Meta-State Analysis Reveals Reduced Resting fMRI Connectivity Dynamism in Schizophrenia Patients. Marinazzo D, ed. *PLoS One*. 2016;11(3):e0149849. doi:10.1371/journal.pone.0149849
